# Analysis of the incidence of false-negative results for SLAP lesions on magnetic resonance imaging

**DOI:** 10.1590/0100-3984.2024.0065-en

**Published:** 2025-05-08

**Authors:** Thiago Bernardo Carvalho de Almeida, João Otávio de Souza Carvalho, Lucas Tonhá de Castro, Eduardo Misao Nishimura, Lucas Bernardo Carvalho de Almeida, Luciano Pascarelli

**Affiliations:** 1 Hospital IFOR – Rede D’Or São Luiz, São Bernardo do Campo, SP, Brazil

**Keywords:** Shoulder injuries/diagnosis, Magnetic resonance imaging, Diagnosis, Data accuracy, Lesões do ombro/diagnóstico, Ressonância magnética, Confiabilidade dos dados

## Abstract

**Objective:**

To evaluate the false-negative rate in the diagnosis of superior labrum anterior to posterior (SLAP) lesions on unenhanced 1.5-T magnetic resonance imaging (MRI).

**Materials and Methods:**

This was a retrospective analysis of the medical records of 24 patients who regularly engaged in physical activity and underwent surgery for reconstruction of the rotator cuff or for glenohumeral instability, comparing the result of the MRI examination with the intraoperative findings.

**Results:**

Eighteen patients (75%) were male and six (25%) were female. False-negative results for SLAP lesions were observed in 83% of the MRI examinations.

**Conclusion:**

For SLAP-type lesions, MRI has low diagnostic sensitivity. Arthroscopy appears to be the most efficient tool for the diagnosis of such lesions.

## Abstract (pt)

**Objetivo:**

Avaliar o índice de falsos-negativos no diagnóstico da lesão SLAP pela ressonância magnética (RM) sem contraste.

**Materiais e Métodos:**

Foram avaliados 24 prontuários de pacientes praticantes de atividades físicas submetidos a cirurgia para reconstrução do manguito rotador ou instabilidade glenoumeral de maneira retrospectiva, comparando o resultado do exame de RM com o relatório cirúrgico.

**Resultados:**

Dezoito (75%) pacientes eram do sexo masculino e seis (25%) eram do sexo feminino. Foram observados 83% de falsos-negativos para lesão de SLAP pela RM de 1,5-T.

**Conclusão:**

O exame de RM 1,5-T apresenta baixa sensibilidade diagnóstica para lesão do tipo SLAP, sendo a artroscopia o meio diagnóstico mais eficiente.

## INTRODUCTION

The investigation of labral lesions, especially in the context of shoulder instability, has been a challenge, particularly in the superior portion of the glenoid labrum and at the insertion of the long head of the biceps. Anatomical studies and surgical findings have facilitated the correlation between clinical findings and lesions in the anteroinferior portion of the labrum. However, the approach to the superior portion of the labrum and the insertion of the biceps has been difficult when classical surgical routes of access have been employed^**(^1^)**^.

It is difficult to obtain well-defined images of the shoulder, even with modern methods such as magnetic resonance imaging (MRI). Therefore, shoulder arthroscopy has come to be highly valued, because it extends the surgeon’s field of vision to include areas that are still little known. This allows injuries that have emerged as “new” in orthopedic knowledge to be diagnosed, evaluated (in static and dynamic studies), and corrected^**(^1^)**^.

A tear involving the superior area of the glenoid labrum, beginning posteriorly and extending anteriorly into the glenoid cavity, is known as a superior labrum anterior to posterior (SLAP) lesion. This area of the glenoid labrum is functionally important for stability of the superior shoulder, serves as an anchor for the insertion of the tendon of the long head of the biceps brachii, and causes significant and often disabling pain when injured, especially in throwing athletes^**(^1^–^3^)**^.

According to a study conducted by Snyder et al.^**(^4^)**^, the incidence of SLAP tears, diagnosed during arthroscopic procedures, is 6%. These lesions are often accompanied by other shoulder injuries, with rotator cuff injuries being the most common, seen in 40% of cases, followed by anterior labrum tears, which are seen in 22%. Clinical and imaging findings have low sensitivity and specificity for the diagnosis of SLAP lesions^**(^1^)**^.

To evaluate cases of shoulder pain, diagnose rotator cuff disease, and identify injury to the long head of the biceps, MRI is widely used and is an accepted method for diagnosing labral lesions. However, for maximum accuracy, the radiologist must be aware of normal anatomic variations of the upper labrum that can complicate correct interpretation. Normal variations, such as a sublabral foramen, a Buford complex (a cord-like middle glenohumeral ligament), a sublabral recess, and a cartilage interface, are well described in the radiology literature.

In this study, we compare the preoperative findings on unenhanced MRI examinations with those obtained during arthroscopic procedures, in the shoulders of athletes. The objective was to evaluate the false-negative rate in the diagnosis of SLAP lesions by unenhanced MRI.

## MATERIALS AND METHODS

This was a cross-sectional observational study with a quantitative approach, conducted from August to December of 2019 at the IFOR Hospital, located in the city of São Bernardo do Campo, Brazil. The project was submitted to the local research ethics committee (Protocol no. CAAE 25851019.0.0000.5625) and was approved (Reference no. 3.807.983). The study followed the ethical guidelines described in Brazilian National Health Council Resolution no. 466/12 and was conducted in accordance with the 1995 Declaration of Helsinki. All participating patients gave written informed consent.

The approach sought to determine the correlation between the images obtained by MRI and the intraoperative findings, as well as to determine the diagnostic accuracy of unenhanced MRI in comparison with that of direct observation of the lesions on arthroscopy.

The study included patients ≥ 18 years of age who engaged in physical activities involving overhead movements, such as volleyball, tennis, jiu-jitsu, boxing, baseball, swimming, functional training, and CrossFit, as well as athletes who underwent surgery at the IFOR Hospital for rotator cuff injuries or anterior glenohumeral instability. All of the patients underwent unenhanced MRI in scanners with a magnetic flux density of at least 1.5 T, and the corresponding radiology reports showed no evidence of SLAP lesions. Patients with other types of shoulder injuries were excluded, as were those who did not engage in physical activities or were not athletes, those for whom the medical records were incomplete or the radiology reports were unavailable, and those whose MRI examinations had significant artifacts, technical problems, or quality insufficient for the correct interpretation of the images, even if there was a corresponding radiology report.

The variables analyzed were age, biological sex, the affected limb, and sports activity, as well as pre-existing comorbidities, such as a history of surgery or extra-articular diseases in the affected shoulder. The data collected were tabulated in Excel spreadsheets for subsequent statistical analysis.

All study participants underwent a preoperative MRI examination in a 1.5-T scanner. The MRI protocol included T2-weighted sequences with fat suppression and T1-weighted fast spin echo sequences acquired in the orthogonal axial, coronal, and sagittal planes, with a field of view of 14 cm and a slice thickness of 4 mm. During the examination, patients were placed in the supine position and entered the machine head first, with the arm to be examined also supinated. This approach sought to ensure the acquisition of detailed, high-quality images of the anatomical structures of the shoulder, with optimal visualization of the lesions, thus facilitating subsequent comparisons with the intraoperative findings.

The MRI images were first analyzed and evaluated by radiologists with extensive experience in shoulder examinations. They were then reviewed by orthopedic surgeons specializing in shoulder injuries, with specific attention to the identification of SLAP lesions. A double-review process was conducted to ensure accurate, comprehensive interpretations of the findings.

To ensure agreement among the interpretations, we held joint discussions in cases with an inconclusive diagnosis, allowing radiologists and orthopedists to compare their interpretations and reach a consensus, especially regarding the presence or absence of SLAP lesions. In cases of divergence between reports, additional meetings were held for discussion among the specialists, with formal recording of the final conclusions, to improve diagnostic accuracy. This qualitative method provided a structured, transparent analysis by consensus, thus promoting a careful, collaborative evaluation of the imaging findings.

The diagnostic criteria used in order to identify SLAP lesions on MRI included the following: an area of high signal intensity, with lateral curvature, in the labrum on a coronal image; multiple or branching lines of high signal intensity in the upper labrum on a coronal image; irregular margins; full-thickness detachment with high signal intensity at the margins, with or without ≥ 2 mm of separation between the labrum and the glenoid cavity on a coronal image; and a paralabral cyst extending from the upper labrum.

The MRI scans and the corresponding radiology reports, as well as the arthroscopy images, were evaluated by three surgeons, all members of the Brazilian Society of Shoulder and Elbow Surgery. Those specialists, with extensive experience in the field, performed a thorough review of the images and reports to ensure an accurate, reliable analysis.

All surgical procedures were supervised by one of the authors of this study. During each shoulder arthroscopy procedure, an intraoperative clinical image (a photograph) was taken with the patient in the beach chair position in a posterior portal view, to determine whether there was a SLAP lesion.

For the arthroscopic diagnosis of SLAP lesions, we followed the criteria proposed by Field et al. and Godinho et al.^**(^5^,^6^)**^: chondromalacia of the superior glenoid; sublabral hemorrhage; separation of ≥ 3 mm between the labrum and the glenoid, when the biceps is under tension; and a bucket-handle tear of the labrum.

We categorized the SLAP lesions in accordance with the classification system devised by Snyder et al.^**(^2^)**^: type I (isolated fraying of the superior labrum with a firm attachment of the labrum to the glenoid—typically degenerative); type II (detachment of the superior labrum and the origin of the tendon of the long head of the biceps brachii from the glenoid, resulting in instability of the biceps-labral anchor); or type III (bucket-handle tear of the labrum with an intact biceps insertion).

### Statistical analysis

The data are presented as means and standard deviations or as absolute frequencies, with or without percentages. Fisher’s exact test was used in order to assess the significance of the occurrence of a SLAP lesion. The assumptions of the hypothesis testing were not compatible with the sample size of type III SLAP lesions^**(^7^)**^. Therefore, our analysis of the proportional distribution of the SLAP lesion types considered only patients with type I lesions or type II lesions. Statistical analyses were performed using GraphPad Prism software^**(^8^)**^. Values of *p* < 0.05 were considered statistically significant.

## RESULTS

Sixty patients were selected for inclusion in the study. Of those, 36 were excluded: 26 because they did not meet the inclusion criteria; and 10 because SLAP lesions were observed on their MRI scans. Therefore, the final sample comprised 24 patients who regularly engaged in physical activity. Of those, 18 (75%) were male and six (25%) were female. The mean age was 33.7 years (range, 18–56 years).

Of the 24 shoulders evaluated, 21 (87.5%) were right shoulders and three (12.5%) were left shoulders. Surgery was performed because of shoulder instability in three patients (12.5%) and because of rotator cuff injury in 21 (87.5%).

**Figure 1:**
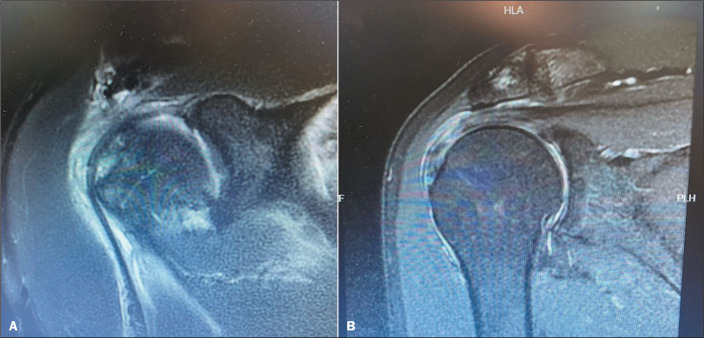
Examples of discrepancy and concordance between MRI findings and intraoperative findings regarding SLAP lesions. **A:** Coronal T2-weighted MRI of the shoulder with a negative radiology report for a SLAP lesion, the presence of which was confirmed intraoperatively. **B:** Coronal T2-weighted MRI of the shoulder with a negative radiology report for a SLAP lesion, the absence of which was confirmed intraoperatively.

Figures 1A and 1B show coronal T2-weighted MRI scans of the shoulder with radiology reports that were both negative for a SLAP lesion. In the first case (Figure 1A), a SLAP lesion was identified intraoperatively, whereas no such lesion was identified in the second case (Figure 1B).

**Figure 2:**
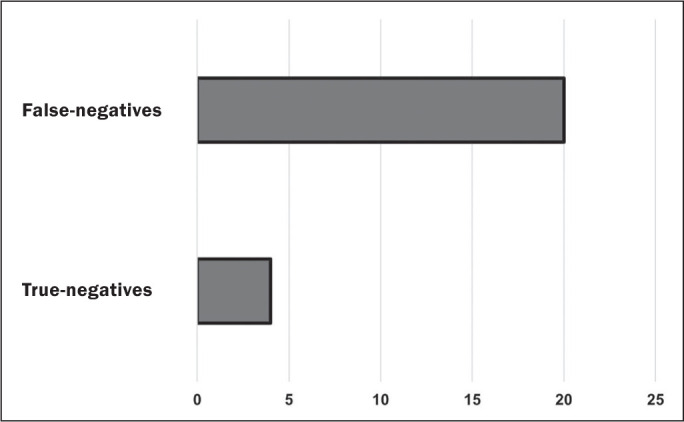
Distribution of patients with and without SLAP lesions, by MRI finding.

**Figure 3:**
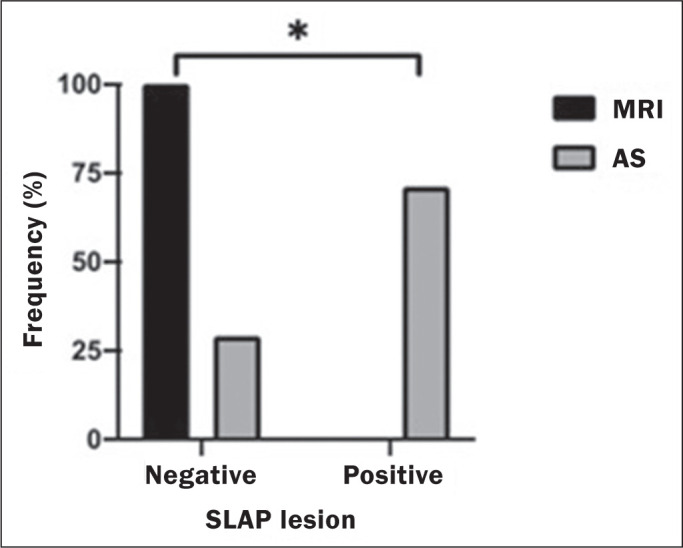
Proportional distribution of a finding of a SLAP lesion by arthroscopic surgery (AS) and MRI. * Significant difference in Fisher’s exact test (*p* < 0.05).

As depicted in Figure 2, true-negative MRI results (radiology report and intraoperative report both negative for a SLAP lesion) were obtained in only four (16.7%) of the 24 cases; in the remaining 20 cases (83.3%), false-negative MRI results (radiology report negative for a SLAP lesion but some type or subtype of SLAP lesion identified intraoperatively) were obtained. As illustrated in Figure 3, the difference between the MRI findings and the intraoperative findings was statistically significant (*p* < 0.001).

**Figure 4:**
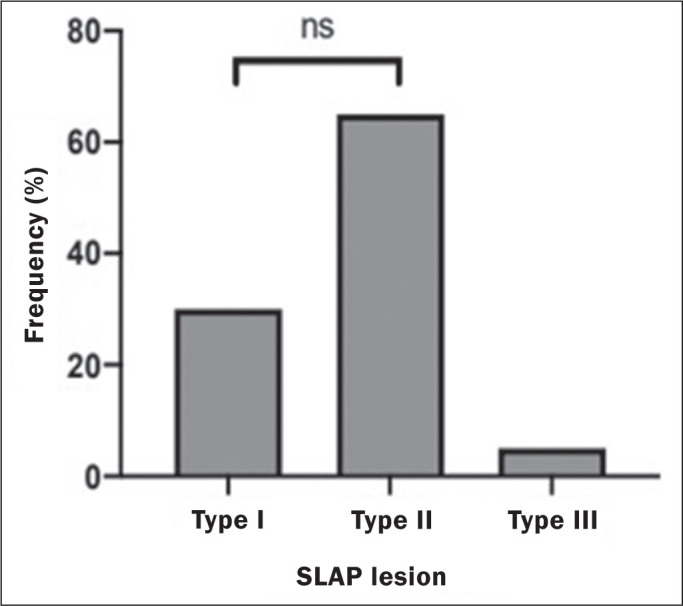
Distribution of SLAP lesion types confirmed by arthroscopy in 19 patients with type I or II SLAP lesions, as well as in one patient with a type III SLAP lesion. ns, Non-significant difference in Fisher’s exact test (*p* > 0.05).

According to the classification by Snyder et al.^**(^2^)**^, the SLAP lesions identified in our study sample were categorized as type I in six patients (30%), type II in 13 (65%), and type III in only one (5%) patient (Figure 4). Despite the different proportions, no significant differences (*p* = 0.242) were observed between types I (32%) and II (68%), indicating there was no significant difference among the types regarding their prevalence.

**Figure 5:**
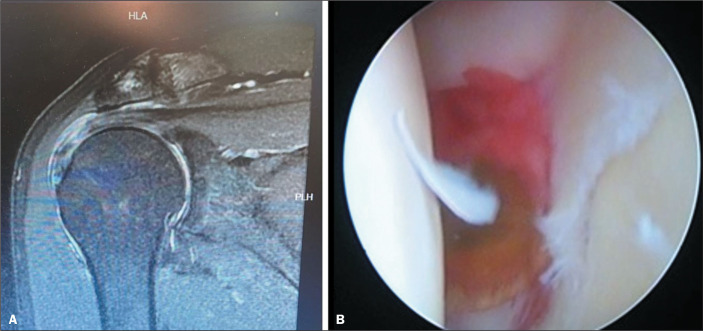
Comparison between MRI and arthroscopy for the diagnosis of a SLAP lesion. **A:** Coronal MRI scan showing a partial tear of the supraspinatus tendon, with no evidence of a labral lesion. **B:** Axial arthroscopy section showing a labral lesion.

Figure 5 presents a comparison between a coronal MRI scan that initially indicated the absence of a SLAP lesion (Figure 5A) and an arthroscopic image of the shoulder of the same patient (Figure 5B) that revealed the presence of such a lesion. This comparison highlights the discrepancy between the results obtained by MRI and direct confirmation of the lesion during arthroscopy.

## DISCUSSION

In throwing athletes, SLAP lesions are a major cause of shoulder pain and imaging is a fundamental diagnostic tool for therapeutic decision-making. According to some studies, MRI has demonstrated a sensitivity of 96% and a specificity of 85% in detecting these lesions^**(^9^,^10^)**^. However, it has not been found to be able to identify age-related degenerative changes. Even diagnostic arthroscopy provides variable results regarding interobserver and intraobserver reliability^**(^11^,^12^)**^.

Houtz et al.^**(^13^)**^ reported that MRI had a sensitivity of 62% and a specificity of 89% for the detection of SLAP lesions. In agreement with that, we found a false-negative rate of 83% in our sample, showing that 1.5-T MRI has low diagnostic sensitivity for the detection of SLAP lesions. In a study conducted in the United States^**(^14^)**^, in which 83 patients with arthroscopically confirmed SLAP lesions were evaluated, MRI showed a sensitivity of 60– 68% and a specificity of 71–77%.

The relationship between the treatment of SLAP lesions and the return to sports has been widely studied. Denard et al.^**(^15^)**^ reported that, at a mean follow-up of 77 months, 87% of patients with arthroscopically repaired type II SLAP lesions had good or excellent results, according to the American Shoulder and Elbow Surgeons and University of California Los Angeles scores. However, in our study sample, 65% of patients with an arthroscopic diagnosis of type II SLAP lesions would probably not have been treated surgically, because the MRI examination did not result in a diagnosis.

Studies in athletes have shown that the sensitivity and specificity of MRI are both 90% for the detection of SLAP lesions^**(^16^,^17^)**^. Such lesions are best detected in oblique coronal sequences, in which the clefts between the labrum and the glenoid are filled with contrast^**(^18^)**^. Various authors have argued that the use of MRI arthroscopy and 3.0-T MRI can increase the accuracy of the diagnosis of rotator cuff injuries^**(^19^–^22^)**^. However, studies have shown no difference when it comes to injuries of the biceps-labral complex^**(^23^,^24^)**^. In addition, many athletes with shoulder overload have labral injuries on examination despite being asymptomatic.

In the present study, involving athletes ≥ 18 years of age, false-negative results for type II SLAP lesions on unenhanced MRI scans were obtained in approximately 83% of the cases, indicating low diagnostic sensitivity. That finding is in contrast with the 6% reported by Snyder et al.^**(^4^)**^. The discrepancy between the two studies could be attributed to patient selection bias. Studies such as that conducted by Snyder et al.^**(^4^)**^ report the incidence of SLAP lesions in a general population undergoing arthroscopy, whereas the present study focused on a specific population of athletes with high functional demands on the shoulder, which could increase the prevalence of SLAP lesions. In addition, the low sensitivity of unenhanced MRI to detect type II SLAP lesions, as demonstrated by Reuss et al.^**(^14^)**^, can result in underdiagnosis in unselected populations.

Our study has some limitations. The retrospective study design could have introduced biases in the selection of data and in the analysis of results, given that the data were collected from existing records, which precludes direct control over important variables and increases the possibility of systematic errors. In addition, the small sample size reduced the generalizability of the findings to larger populations. However, our study also has its strengths. The choice of patients who practice activities involving overhead movements (i.e., athletes in specific sports) brought clinical relevance to the context of rotator cuff injuries and anterior glenohumeral instability, contributing to the practical applicability of the results. In addition, the use of scanners with a magnetic flux density of at least 1.5 T ensured a minimum degree of quality in the images obtained, strengthening the reliability of the analyses performed.

Understanding the early diagnosis of SLAP lesions in athletes is essential, because early detection, together with appropriate treatment, can accelerate the return of the patient to their sport and prevent more serious complications. Early diagnosis and intervention are essential to prevent the progression of the injury and reduce the risk of sequelae, such as functional loss and chronic pain, as well as benefiting physical recovery, maintaining performance, and prolonging the sports career.

## CONCLUSION

In our study sample of adult athletes, unenhanced 1.5-T MRI scans had a false-negative rate of approximately 83% for the detection of SLAP lesions, indicating that they have low diagnostic sensitivity for such lesions.
